# Biparietal meander expansion technique for sagittal suture synostosis in patients older than 1 year of age—technical note

**DOI:** 10.1007/s00381-021-05105-y

**Published:** 2021-03-08

**Authors:** Y. S. Kang, V. Pennacchietti, M. Schulz, K. Schwarz, U-W. Thomale

**Affiliations:** 1 Pediatric Neurosurgery, Charité–Universitätsmedizin Berlin, Campus Virchow Klinikum, Augustenburger Platz 1, D-13353 Berlin, Germany; 2 Neurosurgical Department, Kiel Universitätsmedizin, Kiel, Germany

**Keywords:** Craniosynostosis,, Sagittal synostosis,, Scaphocephaly,, Cranioplasty,, Biparietal meander expansion technique

## Abstract

**Objective:**

Sagittal suture synostosis (SSS) is the most common form of craniosynostosis. For older patients, the strategy for surgical correction needs to consider diminished growth dynamics of the skull and an active reconstruction cranioplasty aims to sustain stability for the active child. We describe our technique of biparietal meander expansion (BME) technique for SSS for patients older than 1 year and retrospectively reviewed the perioperative course as well as the subjective experience of patients and caregivers during follow-up.

**Methods:**

The BME technique incorporates bilateral serpentine craniotomies and fixation of the consecutively expanded bone tongues with crossing sutures for patients with SSS older than 12 months of age at surgery. We reviewed patients undergoing this surgical technique for correction of SSS and collected data about the clinical course and performed a patients reported outcome measure (PROM) for patients or caregivers to evaluate subjective experience and outcome after surgical treatment.

**Results:**

BME was performed in 31 patients (8 females; median age: 43 months; range 13–388). The mean length of operation was 172.7±43 minutes (range 115–294). Patients experienced no immediate complications or neurological morbidity after surgery. Considering a total of 21 completed PROM questionnaires, the head shape after surgery was evaluated as either “better” (57%) or “much better” (43%) compared to preoperatively. Eighty-one percent of patients or caregivers answered that the patient experiences no limitation in daily activities. Although 42.8% perceived the hospital as strenuous, 90.5% would choose to undergo this treatment again.

****Conclusion**:**

BME is a feasible technique for older SSS patients resulting in immediate stability of the remodelled calvarium with a more normal head shape. The survey among caregivers or patients revealed a favourable subjectively experienced outcome after this type of surgical treatment of SSS in the more complex context of an older patient cohort.

## Introduction

Craniosynostosis is a premature fusion of cranial vault sutures, resulting in abnormal head shape and compensatory growth in the region of functionally intact sutures [1, 2]. Sagittal suture synostosis (SSS) is the most common form among the non-syndromic single suture synostosis [3] and shows a strong male predominance with a typical appearance of a dolichocephaly, however, with a wide heterogeneity of phenotypes [3, 4]. In general, aesthetic correction of the skull shape is the indication for surgery, while in some older patients, chronic headaches and speech and language impairment may develop [5–7]. A wide range of different surgical techniques are described in the literature [8–13]. Treatment success correlates mainly with a mouldable thin skull bone together with the ability of dynamic head growth facilitating a semipassive postoperative normalization of the head shape. This ability decreases with age. Thus, in older patients, a total cranial vault remodelling may be necessary.

The current study describes a new technique of biparietal meander expansion (BME) technique, a surgical option for older patients (>12 months) with sagittal synostosis aiming for a stable biparietal expansion.

## Patients and methods

Medical records were retrospectively reviewed for all patients with isolated SSS treated at an age older than 12 months and underwent BME in our institution from 9/2012 to 12/2019. For follow-up evaluation, phone interviews with caregivers or patients were performed for patient reported outcome measures (PROM). The study was approved by the institutional ethics committee (EA2/003/16).

**Fig. 1:**
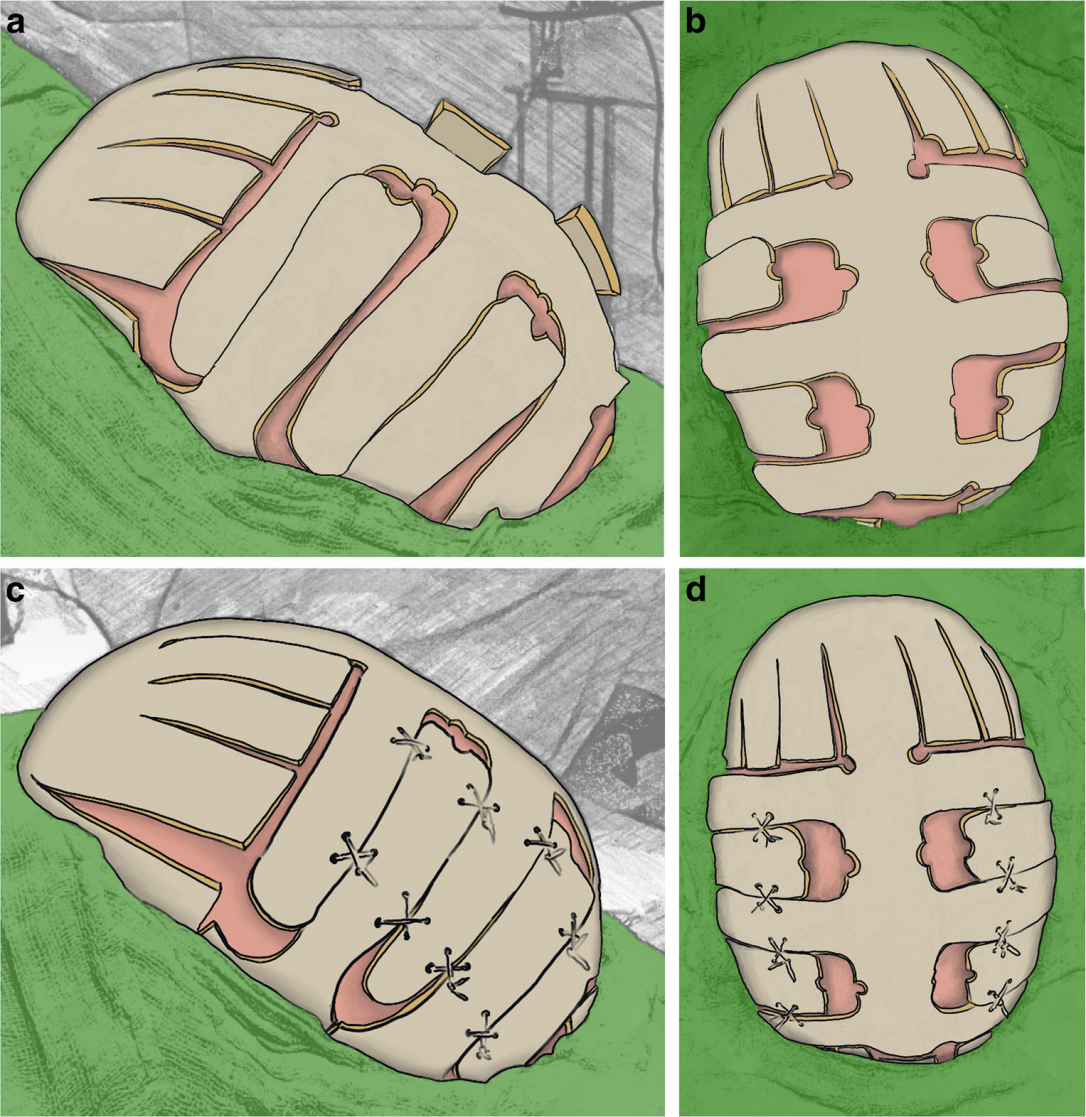
Intraoperative schematic pictures displaying the application of meander shape craniotomies bilaterally as well as barrel stave incisions frontally and occipitally. **a** Lateral view, **b** view from above. After application of crossing sutures, the bone tongues are distracted to each other and fixed resulting in a biparietal expansion of the convexity and elevation of the vertex. In addition, the barrel stave bone strips in the frontal and occipital region are elevated to the level of biparietal expansion, accordingly. **c** Lateral view, **d** view from above

The *surgery* (Fig. 1) was performed under general anaesthesia and the patient was placed in supine position. Strip-shaped hair shaving in bicoronal fashion, disinfection and draping are performed. A curved bicoronal skin incision enabled the preparation of an anterior and posterior galeal flap to expose the coronal and lambdoid sutures. Biparietal meander lines are marked on the skull from paramedian to the temporal area of the calvarium placing burr holes at the paramedian tip of the markings. Craniotomies are performed along the meander lines bilaterally connecting each burr hole. The resulting intersecting bone tongues are based reciprocally at the midline or temporal calvarium. Just in front of lambda, the sagittal bone strip is transected across the midline. The underlying dura is dissected from the tongues from the midline vertex strip over the sinus and emissary veins are coagulated. In the temporal region, the bone incisions are extended further towards the cranial base crossing the squamous sutures. Barrel stave bone incisions are applied with a length of about 4cm frontally and occipitally perpendicular to the last meander line crossing the lambdoid and coronal sutures, respectively. The biparietal bone tongues are distended against each other and fixed with crossing sutures to achieve biparietal expansion, which also lifted the vertex. Applying an additional suture from the sagittal bone to the occipital midline edge, the vertex is adapted for optimal sagittal contouring. The barrel-shaped bone strips are elevated and fixed with sutures for adaptation of the height at the frontal and occipital edges. A subgaleal drainage tube is placed and skin flaps are repositioned and closed by subcutaneous sutures and by either skin glue (Dermabond, Ethicon, J&J, USA) or monofilament sutures (Prolene 3-0, Ethicon, J&J, USA).

## Results

**Fig. 2:**
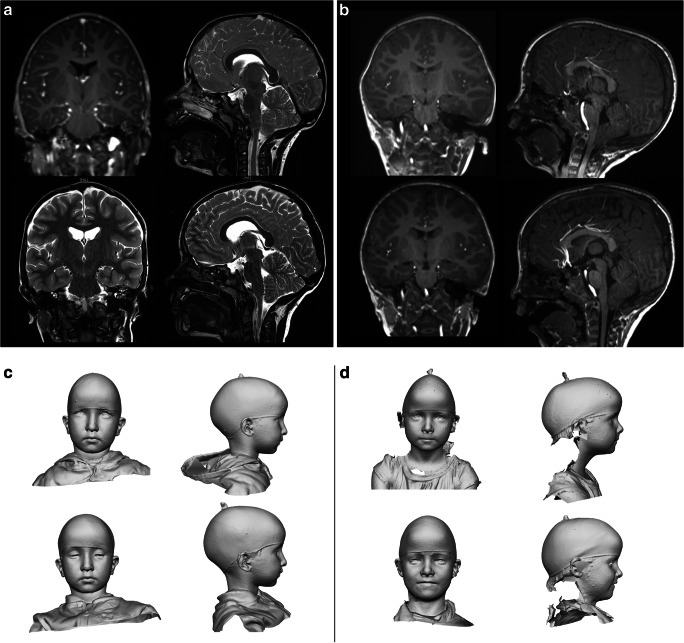
Representative MRI from patients receiving BME surgical procedure for SSS. **a** 5-year-old boy with sagittal suture synostosis showing biparietal narrowing in the coronal section and tight external CSF spaces in the sagittal section preoperatively (upper left row), which resulted in significant parietal widening and a release of external CSF spaces are shown in the MRI at 5 months after surgery (lower left row). **b** 3-year-old boy with SSS showing bilateral parietal narrowing in the coronal section and a scaphocephalic descending vertex with secondary Chiari malformation in the sagittal section preoperatively (upper right row). A widened and smooth coronal circumference and elevation of the vertex as well as improvement of the Chiari malformation is shown postoperatively at 9 months after surgery (lower right row). Representative 3D photography from patients receiving BME surgical procedure for SSS. **c** 6-year-old boy showing a typical scaphocephalic head shape with narrowing in the anterior view and a elongation with parieto-occipital sharpening in the side view preoperatively (upper left row). After surgery, a parietal expansion and a smoother curvature of the convexity are achieved in both views (lower left row). **d** 5-year-old girl with marked biparietal narrowing in the anterior view and sagittal elongation with decreased vertex slope and secondary frontal bossing preoperatively (upper right row). Postoperatively, the parietal expansion can be visualized in the anterior view and a normalized sagittal curvature with an elevated vertex and less pronounced bossing can be appreciated in the lateral view (lower right row)

In thirty-one patients (23 males) with a mean age of 83.4±97 months (range: 13–388), the BME technique was successfully applied (Fig. 2). All data of the perioperative course are given in Table 1.

**Table 1:** Patient characteristics

*n*	31	
Sex	23 males/8 females	
Comorbidities	Hypophosphatism (*n*=2) Pilocytic astrocytoma (*n*=1) Di George immunopathy syndrome (*n*=1) Alopecia universalis (*n*=1)	
	Mean±STD	Range
Age	83.4±97 months	13–388 months
Length of surgery	172.7±43.2 min	115–294 min
ICU stay	2±0.85 days	1–4 days
Hospital stay	6±1 days	4–8 days
PreOP haemoglobin	12.5±1.4 g/dl	10.2–16.4 g/dl
PostOP haemoglobin	7.9±1.6 g/dl	5.7–13.2 g/dl
Transfusion rate	64.5% (20/31)	
Packed red blood cell transfusion	0.84±0.7 per patient	0–2 per patient(*n*=2 in 6 patients)
Complication	Seizure (*n*=1) without intracranial haemorrhage or oedema in MRI	
Follow-up time	1.9±1.6years	0.3–7.4years
Follow-up surgeries	Chiari decompression (*n*=1) Telemetric ICP (*n*=1) Redo BME (*n*=1) after 5 years	

**Fig. 3:**
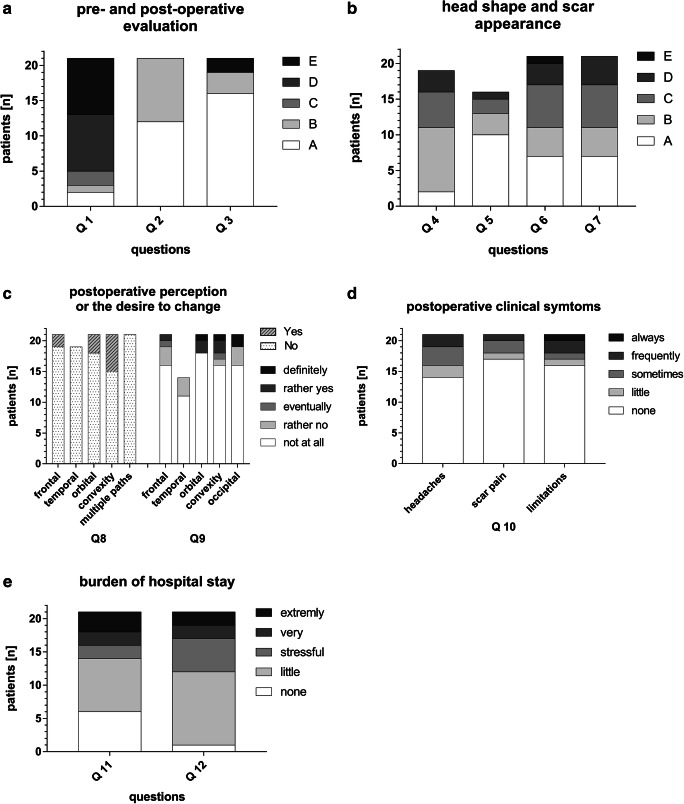
Patient reported outcome questionnaire. **a** General evaluation of preoperative and postoperative appearance as well as general experience of treatment. *Q1.* How would you rate head deformity experienced before the operation? *Answers*: A: not obvious 9.5%, B: little obvious 4.8%, C: obvious 9.5%, D: very obvious 38.1%, E: extremely obvious 38.1%. *Q2.* How would you rate the postoperative head shape compared to before the operation? *Answers*: A: much better 57.1%, B: better 42.9%, C: same 0%, D: worse 0%, E: much worse 0%. *Q3.* Based on your current knowledge and experience, would you choose this treatment again? *Answers*: A: yes, in any case 76.2%, B: yes 14.3%, C: eventually 0%, D: no 0%, E: not at all 9.5%. **b** *Q4.* How would you rate your/the current head shape /of your child? *Answers:* A: excellent 10.5%, B: very good 47.4%, C: good 26.3%, D: acceptable 15.8%, E: inacceptable 0%. *Q5.* Has ever someone mentioned your / your child’s head shape after the operation? *Answers:* A: never 62.5%, B: almost never 18.8%, C: sometimes 12.5%, D: frequently 6.3%, E: almost always 0%. *Q6.* How would you evaluate your/ the scar on your / child’s head? *Answers:* A: excellent 33.3%, B: very good 19%, C: good 28.6%, D: acceptable 14.3%, E: inacceptable 4.8%. *Q7.* Has ever someone mentioned to you the noticeable scar on your / child’s head? *Answers:* A: never 33.3%, B: almost never 19%, C: sometimes 28.6%, D: frequently 19%, E: almost always 0%. **c** *Q8***.** When someone mentioned to you your/child’s head shape, which part of the head did they address? *Answers:* A: frontal region, no 90.5%, yes 9.5%; B: temporal region, no 100%, yes 0%; C: orbital region, no 85.7%, yes 14.3%; D: convexity, no 71.4%, yes 28.6%; E: multiple areas, no 100%, yes 0%. *Q9***.** If you would have a chance to correct your child’s head shape, which part would you change? *Answers:* not at all – frontal: 76%, temporal: 79%, orbital: 86%, convexity: 76%, occipital: 76%; rather no – frontal: 14%, temporal: 21%, orbital: 0%, convexity: 5%, occipital: 14%; eventually – frontal: 5%, temporal: 0%, orbital: 0%, convexity: 5%, occipital: 0%; rather yes – frontal: 5%, temporal: 0%, orbital: 10%, convexity: 10%, occipital: 0%; definitely – frontal: 0%, temporal: 0%, orbital: 5%, convexity: 5%, occipital: 10%. **d** *Q10*. Does your child have headache? *Answers*: none: 66.7%, little: 9.5%, sometimes: 14.3%, frequently: 9.5%, always: 0%; Does your child have scar pain? *Answers*: none: 81%, little: 4.8%, sometimes: 9.5%, frequently: 4.8%, always: 0%; Does your child have any limitation in daily activities? *Answers*: none: 76.2%, little: 4.8%, sometimes: 4.8%, frequently: 9.5%, always: 4.8%. **e** *Q11*. How strenuous would you rate the hospital experience for yourself? *Answers*: none 28.6%, little 38.1%, stressful 9.5%, very 9.5%, extremely 14.3%. Q12. How strenuous would you rate the hospital stay for your child? *Answers*: none 4.8%, little 52.4%, stressful 23.8%, very 9.5%, extremely 9.5%

The postoperative PROM outcome was evaluated in 21 patients (14 males, mean age: 5.4±5.5 years, range: 1.1–17.7years) by either three patients (>10 years of age) or 18 caregivers (follow-up time: 1.9±1.6 years; Fig. 3).

## Discussion

According to our experience, the BME technique is feasible and safe for older patients with SSS. The perioperative hospital stay and the amount of blood loss during surgery remain in the expected range in relation to the treatment intensity. Complications of cranial vault reconstruction such as dural tears, CSF leak, hematoma, impaired wound healing or infection are observed [14]. One patient suffered from a postoperative seizure without any oedema or haemorrhage in immediate MRI. It is reported that surgical correction of craniosynostosis may involve significant blood loss [15, 16], 64% received a blood product transfusion while 19% received two transfusions. No sinus injury was observed in our series. According to our observation, the skin incision, the spongiosa of the calvarium and the emissary veins of the dura are the main source of blood loss. Once the bone segments are developed and thereby calvarial constriction is released, bleeding is normally well controlled.

Since the indication for surgery is mainly aesthetic due to psychological impairment caused by the abnormally experienced head shape, the evaluation of a successful surgery remains to be subjective [2, 17]. The postoperative follow-up by patient reported outcome measures (PROM) reflects an overall satisfaction after BME; however, almost half of the patients rated the hospital stay as “stressful”. All of the patients judged the cosmetic result of the surgery to be “good” or “very good”. PROM may further become an important instrument to assess the postoperative situation in craniosynostosis patients [18].

Controversy persists about headaches being caused by increased ICP in SSS patients [7]. Two series of intraoperative monitoring in SSS patient revealed elevated ICP values being 16.1±2.4 mmHg mainly in infants or higher than 20cm H_2_O in 82% of older patients [19, 20] underlining the need for volume expansion.

We personally evaluate the BME technique to achieve a decent biparietal widening with relevant gain of intracranial volume in combination with good calvarial stability. Thus, none of the patients experienced any traumatic incident leading to complaint concerning the result of surgery. The main disadvantage of the technique may be that it focuses mainly on the biparietal and temporal region of calvarium. As adaptation of the technique forehead remodelling may be additionally suggested in patients with relevant frontal bossing [12].

We conclude that BME technique in patients with sagittal synostosis at older age offers feasible biparietal widening with decent stability towards a normalization of the head shape. Future investigation should further focus on quantitative outcome measures such as 3D photography.
